# Smoking correlates with increased cytoskeletal protein‐related coding region mutations in the lung and head and neck datasets of the cancer genome atlas

**DOI:** 10.14814/phy2.13045

**Published:** 2016-12-30

**Authors:** John M. Yavorski, George Blanck

**Affiliations:** ^1^ Department of Molecular MedicineMorsani College of MedicineUniversity of South FloridaTampaFlorida; ^2^ Immunology ProgramH. Lee Moffitt Cancer Center and Research InstituteTampaFlorida

**Keywords:** Cytoskeleton, DNA methylation, extracellular matrix, mutation, oncogenes, smoking cessation, TCGA, tobacco use, tumor suppressor genes

## Abstract

Cancer from smoking tobacco is considered dependent on mutagens, but significant molecular aspects of smoking‐specific, cancer development remain unknown. We defined sets of coding regions for oncoproteins, tumor suppressor proteins, and cytoskeletal‐related proteins that were compared between nonsmokers and smokers, for mutation occurrences, in the lung adenocarcinoma (LUAD), head and neck squamous carcinoma (HNSC), bladder carcinoma (BLCA), and pancreatic adenocarcinoma ( PAAD) datasets from the cancer genome atlas (TCGA). We uncovered significant differences in overall mutation rates, and in mutation rates in cytoskeletal protein‐related coding regions (CPCRs, including extracellular matrix protein coding regions), between nonsmokers and smokers in LUAD and HNSC (*P* < 0.001), raising the question of whether the CPCR mutation differences lead to different clinical courses for nonsmoker and smoker cancers. Another important question inspired by these results is, whether high smoker cancer mutation rates would facilitate genotoxicity or neoantigen‐based therapies. No significant, mutation‐based differences were found in the BLCA or PAAD datasets, between nonsmokers and smokers. However, a significant difference was uncovered for the average number of overall cancer mutations, in LUAD, for persons who stopped smoking more than 15 years ago, compared with more recent smokers (*P* < 0.032).

## Introduction

Tobacco use has long been associated with increased incidence of lung (Kasala et al. 2015), head and neck (Hayes et al. 2015), pancreatic (Maisonneuve and Lowenfels 2015) and bladder cancer (Cumberbatch et al. 2016), but the mechanisms of cancer initiation and progression dependent on tobacco use are not comprehensively understood. In particular, mutations caused by smoke‐related mutagens are considered an important cause (Kim et al. 1993), but other factors, such as smoking dependent, reduced CpG island methylation, could also play a role (Klingbeil et al. 2014; Philibert et al. 2014). We grouped nonsmokers and smokers using the TCGA clinical files and compared their mutation occurrences. The analysis of the data below indicates that recognized cancer driver mutations in lung adenocarcinoma distinguish nonsmokers from smokers but not in head and neck, bladder or pancreatic cancer. In bladder cancer, in particular, our analysis indicates that lower methylation (and thereby reduced suppression) of oncogenes in smokers may be the distinguishing factor between nonsmoker‐ and smoker‐related disease.

Cytoskeletal proteins are emerging as a distinct class of cancer driver proteins, particularly in view of their likely capacity for function as dominant‐negative drivers (Fawcett et al. 2015; Parry et al. 2015; Parry and Blanck 2016), in that mutations in any one of many places in the coding region of a filamentous protein would have the impact of disrupting polymer formation, as happens with any number of mutations in the coding regions for the collagen subunits that form collagen molecules and cartilage. In addition, cytoskeletal‐related protein coding regions (CPCRs), including extracellular matrix (ECM) protein coding regions, occupy a relatively large genomic space. For example, the CPCR set that is among the top 25 most mutated coding regions among 10 cancer datasets represents about 15% of all of the human coding regions (Parry and Blanck 2016). Thus, this CPCR set has great potential as a biomarker of mutagenesis. This is particularly true keeping in mind the many reagents and processes available to assess cytoskeletal and ECM integrity and the fact that a disorganized cytoskeleton has been long associated with tumorigeneis (Verderame et al. 1980). Thus, this report includes an assessment of mutation rates for this previously established, cancer‐relevant CPCR set.

## Methods

Clinical and somatic mutation data (BI_Illumina) were downloaded for the lung adenocarcinoma (LUAD), head and neck squamous carcinoma (HNSC), bladder carcinoma (BLCA), and PAAD datasets from the TCGA data portal (http://cancergenome.nih.gov/), following NIH approval (request #27073‐3 for project #6300). The clinical patient file was used to sort barcodes into lifelong nonsmokers and smokers for each cancer dataset. Tumor sample barcodes in the somatic mutation file were truncated to contain only the following characters, TCGA‐##‐####. A cancer file, for example, “SOM, LUAD results”, was created for each cancer set. The truncated tumor sample barcodes along with the nonsmoker and smoker barcodes were then copied into the total mutations sheet of each cancer file. A COUNTIF function was used to determine the total number of mutations for each barcode in both the nonsmoking and smoking categories. Barcodes in nonsmoker and smoker categories, respectively, that did not appear in the mutation file were eliminated. The final lists of nonsmoker and smoker barcodes representing mutations were determined. The nonsmoking and smoking barcodes were then compared based on their total mutation frequencies.

Three sets of coding regions, for analysis of mutation occurrence between nonsmoker and smoker categories, were established: (1) cytoskeletal protein‐related coding regions (CPCRs) (Fawcett et al. 2015; Parry and Blanck 2016; Parry et al. 2015); (2) oncoprotein (Fawcett et al. 2015; Parry et al. 2015); (3) tumor suppressor proteins (Fawcett et al. 2015; Parry et al. 2015). The CPCRs were previously determine in ref. (Parry et al. 2015), based on their occurrence in the top 25 most commonly mutated coding regions among the five TCGA datasets studied in ref. (Parry et al. 2015) (BLCA, COAD, LUAD, GBM, STAD). Three additional CPCRs were added to the set indicated in the preceding sentence, representing commonly mutated CPCRs in the SKCM dataset. The HUGO symbols for the coding regions for all sets are in Table 1.

**Table 1:** HUGO symbols for the CPCR, oncoprotein, and tumor suppressor protein gene sets (detailed in the SOM file labeled, “SOM Table 1, source file”)

Gene set				
Cytoskeletal		Oncoprotein	Tumor suppressor	
ANK2	MUC4	ACVR1	AKAP12	KISS1R
APC	NEB	ALK	AXIN1	KLF6
COL11A1	NEFH	ARAF	BMP2	LATS2
DNAH10	NF1	BRAF	BMPR1B	LIMD1
DNAH11	PCDH15	CTNNB1	BMPR2	MAP2K4
DNAH3	PCDHAC2	EGFR	BRCA1	MED23
DNAH5	PCDHGC5	FGFR2	BRCA2	PBRM1
DNAH7	PCLO	FLT3	BRMS1	PEBP1
DNAH8	PKHD1	FRK	CASZ1	PPAPDC1B
DSCAM	PLEC	HRAS	CDKN2A	PRDM2
DST	RELN	JAK2	CHD5	PTEN
FAT3	SPTA1	KRAS	CHEK2	RB1
FAT4	SPTAN1	MTOR	CTCF	RECK
FBN2	SSPO	NRAS	DLC1	SMAD4
FLG	SYNE1	PRKACA	DOK2	SMAD7
GPR98	SYNE2	RAF1	FLCN	SMARCB1
MUC16	TTN		FOXP3	SP100
MUC17	XIRP2		GPR68	TFPI2
			ING1	TMPRSS11A
			ING4	TXNIP
			INPP4B	VHL
			KISS1	WWOX

CPCR, cytoskeletal protein‐related coding regions.

Within each cancer file (e.g., “SOM, LUAD results”), a sheet for total mutations, including only the original barcodes truncated as indicated above and two sheets for each coding region set, for example, “CPCR Mut.” and “CPCR Results,” were created. The coding region mutation sheet includes information from the comprehensive mutation file, such as HUGO symbol, truncated tumor sample barcode, and mutation type (amino acid altering or silent). The list of nonsmoker and smoker barcodes was compared to the comprehensive mutation data using a COUNTIF function (in the coding region mutation sheet) to determine the number of mutations per barcode. The coding region mutations of nonsmokers and smokers were compared in the “results” sheet.

From the LUAD clinical patient file, available data on the years started and stopped smoking were collected (“SOM Table 2, source file”). Groups of (1) less than or equal to, (2) greater than 15 years from the time of smoking cessation, (3) less than or equal to, and (4) greater than 30 years of total years of smoking were established and then compared based on the number of total mutations per barcode.

**Table 2:** Comparison of available data from the LUAD dataset based on the year started and stopped smoking (detailed in the SOM file labeled, “SOM Table 2, source file”)

	Number of years since stopped smoking (≤15 years vs. >15 years)		Total number of years smoked (≤30 years vs. >30 years)
*P*‐value for ≤15 years versus >15 years since stopped smoking	0.0315	*P*‐value for ≤30 years versus >30 years of smoking	0.9148
Avg number of mutations/barcode for ≤15 years	604.06	Avg number of mutations/barcode for >30 years	493.11
Avg number of mutations/barcode for >15 years	387.65	Avg number of mutations/barcode for ≤30 years	482.55

LUAD, lung adenocarcinoma.

The number of deleterious amino acid changes was determined using PROVEAN. The chromosome number, start position, reference allele, and tumor sequence allele for the CPCR and tumor suppressor datasets for nonsmokers and smokers in each cancer were copied and pasted into PROVEAN under the Human Genome Variants protocol. The removal of duplicates from the “#ROW_NO.” column in the PROVEAN output was used to determine the number of deleterious amino acid changes for the CPCR and tumor suppressor datasets. The total was then divided by the sample size to determine the average number of deleterious amino acid changes for nonsmokers and smokers in each cancer set. (An example with additional detail is provided in the SOM file labeled, “SOM Example Deleterious AA, LUAD CPCR”).

Level 3 JHU‐USC HumanMethylation450 data for the top five available barcodes with the highest mutation burdens for nonsmokers and smokers of each cancer set were downloaded from the TCGA data portal. Average beta values for the entire methylation file and oncoprotein datasets were determined for each barcode (“SOM Table 4, source file”).

## Results

**Figure 1:**
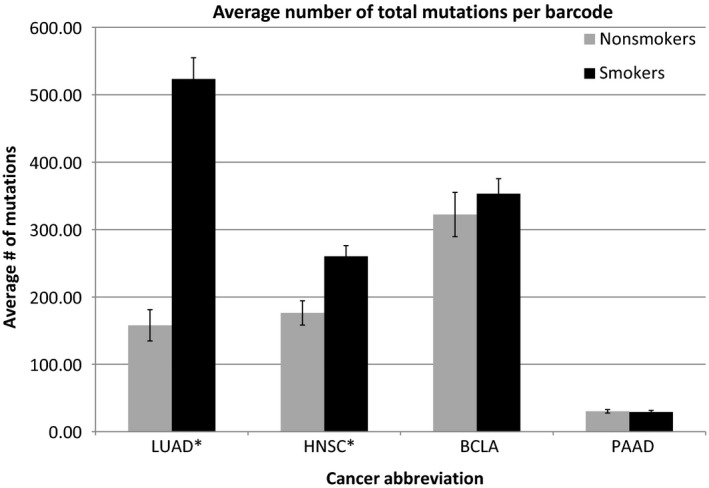
Average number of total mutations per barcode for nonsmokers versus smokers for each cancer type. *indicates *P* < 0.001 (LUAD *P* = 1.16e^‐18^) (HNSC *P* = 0.00057) (BLCA and PAAD *P* > 0.05 not significant) (detailed in the SOM file labeled, “SOM Figs. 1 and 2, source file”). Demographic information for all four of the indicated cancer datasets: Gender, race, and age were recovered from the TCGA clinical files for the LUAD, HNSC, BLCA, and PAAD cancer sets for nonsmokers and smokers (detailed and summarized in distinct sheets of the Excel SOM file labeled, “SOM Demographics”). There were no significant differences in the age ranges for nonsmokers versus smokers in any of the cancer sets. The vast majority of the subjects were white, and no conclusions can be drawn from the race/ethnicity groupings. Finally, there was a slight skewing of more males, on a percentage basis, who were smokers, for each cancer dataset, but again, the differences were not sufficient for a statistical analysis. BLCA, bladder carcinoma; PAAD, pancreatic adenocarcinoma.

The TCGA datasets of LUAD, HNSC, BLCA, and PAAD were compared based on mutation frequencies between lifelong nonsmokers and smokers. The total number of mutations per barcode was calculated, and the average number of mutations per barcode was determined for nonsmokers and smokers for each cancer type (Fig. 1). The overall mutation rates for LUAD and HNSC, distinguishing nonsmokers and smokers, were found to be statistically significant, however, no such distinction could be established for the BLCA and PAAD datasets.

**Figure 2:**
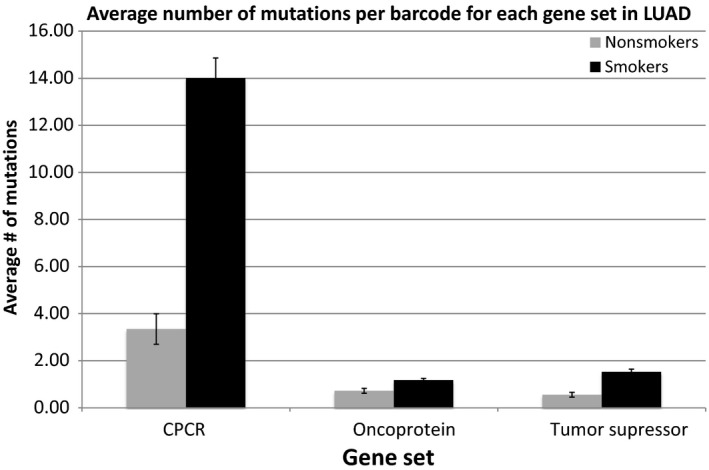
Average number of mutations per barcode for each gene set in LUAD for nonsmokers versus smokers. All gene sets have *P* < 0.001 (detailed in the SOM file labeled, “SOM Figure 1 and 2, source file”).

**Figure 3:**
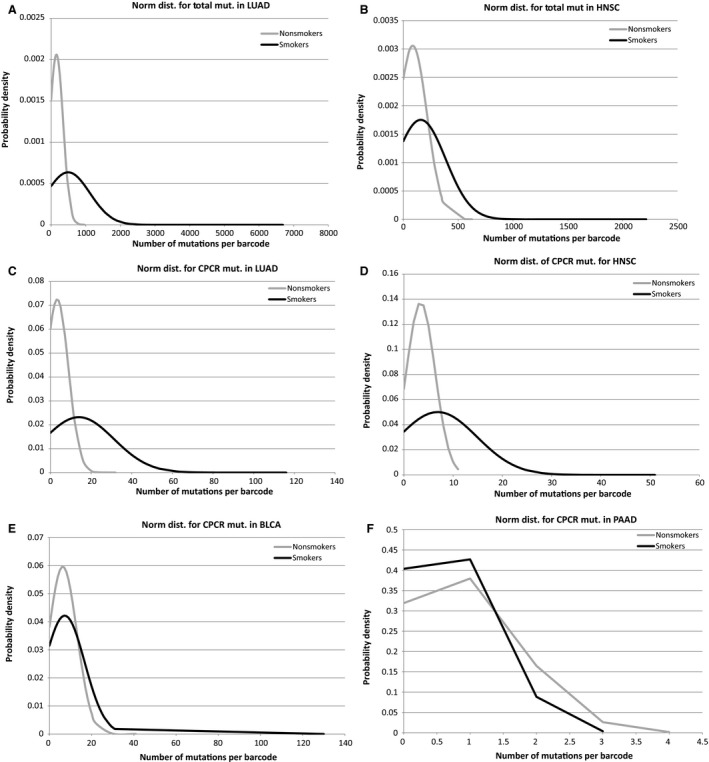
(A) Normal distribution of total mutations per barcode in LUAD for nonsmokers versus smokers. (*P* < 1.0e^‐17^) (detailed in the SOM file labeled, “SOM, LUAD results”). (B) Normal distribution of total mutations per barcode in HNSC for nonsmokers versus smokers. (*P* < 0.0006) (detailed in the SOM file labeled, “SOM, HNSC results”). (C) Normal distribution of CPCR mutations per barcode in LUAD for nonsmokers versus smokers. (*P* < 1.3e^‐20^) (detailed in the SOM file labeled, “SOM, LUAD results”). (D) Normal distribution of CPCR mutations per barcode in HNSC for nonsmokers versus smokers. (*P* < 1.5e^‐7^) (detailed in the SOM file labeled, “SOM, HNSC results”). (E) Normal distribution of CPCR mutations per barcode in BLCA for nonsmokers versus smokers. (*P* > 0.05 not significant) (detailed in the SOM file labeled, “SOM, BLCA results”). (F) Normal distribution of CPCR mutations per barcode in PAAD for nonsmokers versus smokers. (*P* > 0.05 not significant) (detailed in the SOM file labeled, “SOM, PAAD results”). CPCR, cytoskeletal protein‐related coding regions; HNSC, head and neck squamous carcinoma; LUAD, lung adenocarcinoma; PAAD, pancreatic adenocarcinoma.

Next, three cancer driver gene subsets (Fawcett et al. 2015; Parry et al. 2015) were established for analysis: (1) CPCRs; (2) oncoprotein; (3) tumor suppressor proteins (Table 1). Mutation frequencies were evaluated for nonsmokers and smokers for each gene set for the four cancer types. Smokers had a significantly higher average mutation rate for all three coding region sets for LUAD (Fig. 2); and smokers had a significantly higher number of mutations for the CPCR gene set in HNSC (Fig. 3), reflecting the HNSC results for the overall mutation rates and the distinction between nonsmokers and smokers (Fig. 1). Mutations rates of the remaining gene sets for HNSC (oncoprotein and tumor suppressor protein) failed to distinguish nonsmokers and smokers in the indicated analyses. None of the gene set mutation rates distinguished nonsmokers from smokers for the BLCA and PAAD datasets.

To further emphasize the distinction in mutation rates, between nonsmokers and smokers, for LUAD and HNSC, the 25 barcodes with the highest mutation frequency and the 25 barcodes with the lowest mutation frequency were compared based on their inclusion in the nonsmoker or smoker groups (detailed in the SOM files labeled, “SOM, LUAD results” and “SOM, HNSC results”). In both cases, the high‐frequency mutation groups were dominated by smoker barcodes (LUAD *P* < 2E^‐12^, HNSC *P* < 6E^‐8^) and the low‐frequency mutation groups were about evenly distributed between nonsmokers and smokers.

**Figure 4:**
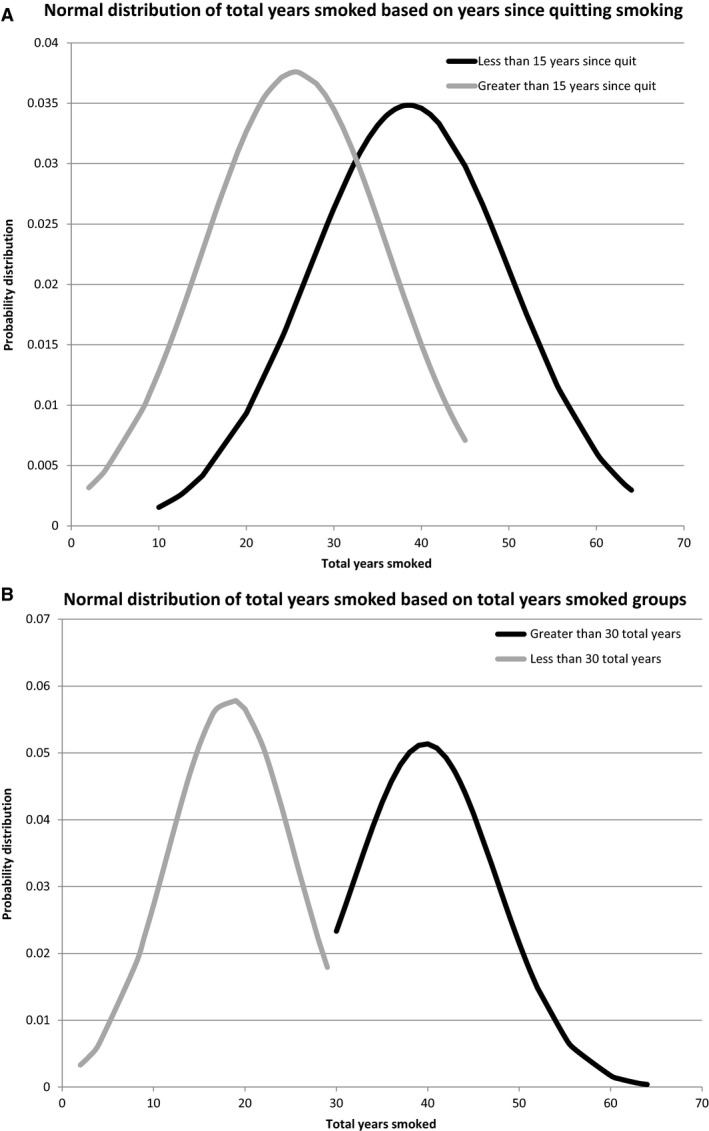
(A) Normal distributions for the total years smoked between the ≤15 years since quitting and >15 years since quitting smoking groups (detailed in the SOM file labeled, “SOM Table 2, source file”). (B) Normal distributions of total years smoked between the ≥30 years and <30 years of total years smoking (detailed in the SOM file labeled, “SOM Table 2, source file”). BLCA, bladder carcinoma; HNSC, head and neck squamous carcinoma.

Public health data have strongly indicated, for decades, that smoking cessation is accompanied by a reduced risk of cancer and the results continue to be confirmed with recent work (Wynder and Hoffmann 1976; Wynder and Stellman 1977; Ogihara et al. 2016). However, possible molecular explanations for this phenomenon have been limited (Wang et al. 1999), usually to specific genes. We found that groups of (1) less than or equal to, and (2) greater than 15 years since smoking cessation had a significant difference in the number of mutations per barcode. Barcode groups of (3) less than or equal to, and (4) greater than 30 years of total years smoked had no difference (Table 2), indicating that the significant difference in the number of mutations between the smoking and smoking‐cessation groups was not simply due to fewer years smoked among the smoking‐cessation group. To further address this (trivial) possibility, we examined the distribution of the total number of years smoked in all of the above‐indicated sets, and it is clear that the smoking and smoking cessation groups have a much larger overlap of years smoked than do the two groups based on total number of years smoked (Fig. 4). This strongly indicates that the significant difference in the number of mutations between the smoking and smoking‐cessation groups is not due to a difference in the number of mutations accumulating on a per‐year basis. If that were the case, the difference in the number of mutations distinguishing the “over 30 years” and under 30 years groups would likely be significant, that is, parallel to the average number of years smoked. (In this latter case, the average number of years smoked is dramatically different based on the distributions of years smoked (Fig. 4B), but the number of mutations in the cancer samples is not (Table 2).)

To address whether the higher smoker mutation rates could have an impact on cellular function, the number of deleterious amino acid changes for the CPCR and tumor suppressor datasets were also considered (Table 3). The LUAD dataset had the largest average number of deleterious amino acid changes per barcode, as well as, the greatest difference between nonsmokers and smokers in both the CPCR and tumor suppressor datasets, consistent with the above distinctions between nonsmokers and smokers based on mutations alone.

**Table 3:** Average number of deleterious mutations per barcode in the CPCR and tumor suppressor data sets for all four cancer sets (detailed in the SOM file labeled, “SOM Table 3, source file” and additional information for the method is detailed in the SOM file labeled, “SOM Example Deleterious AA, LUAD CPCR”)

	LUAD		HNSC		BLCA		PAAD	
	Smokers	Nonsmokers	Smokers	Nonsmokers	Smokers	Nonsmokers	Smokers	Nonsmokers
Avg # of deleterious CPCR mut/barcode	4.43	0.86	2.28	0.90	2.15	1.83	0.19	0.19
Avg # of deleterious tumor suppressor mut/barcode	0.52	0.15	0.45	0.36	0.37	0.50	0.09	0.06

BLCA, bladder carcinoma; CPCR, cytoskeletal protein‐related coding regions; HNSC, head and neck squamous carcinoma; LUAD, lung adenocarcinoma; PAAD, pancreatic adenocarcinoma.

Data indicated that both BLCA and PAAD development are associated with smoking. Likewise, HNSC has been linked to smoking. Yet, in none of these cases were we able to observe oncoprotein or tumor suppressor protein mutation increases among the smokers. There are numerous possible explanations for this, not the least of which is that the difference between nonsmokers and smokers is related to incidence of occurrence of a “single hit” basis for the cancers and not due to any “amplification” requirement that could be traceable to an increased number of mutations. Nevertheless, the increased number of mutations in cancer driver coding regions in the LUAD dataset raises the question of whether nonsmokers could be distinguished from smokers via another molecular attribute related to cancer development.

Thus, we considered the possibility that nonsmokers and smokers could be distinguished on the basis of reduced methylation of oncoprotein coding regions, presumably leading to a relative upregulation of oncoprotein expression. Indeed, there have been reports linking smoking chemicals to interference in CpG island methylation (Klingbeil et al. 2014; Philibert et al. 2014; Bjaanaes et al. 2016). Thus, we compared the methylation averages for the top five available barcodes with the highest mutation burdens for nonsmokers and smokers in each cancer set (Table 4). We selected the barcodes with the highest numbers of mutations to effect the premise that, in demonstrating a nonmutation possibility for the mechanism of the smoking impact, the highest standard would be such a demonstration where samples had many mutations. This is in contrast to a comparison of samples with few or no mutations (e.g., no mutations in oncoproteins) where a second mechanism must be in effect. In other words, a second mechanism should be identifiable above a relatively high background of mutations, because for certain gene sets and cancer datasets, detailed above, mutation rates do not distinguish nonsmokers from smokers.

**Table 4:** Methylation beta averages for the top five available barcodes with the highest mutation burdens for smokers and nonsmokers in all four cancer sets for the overall genome methylation and the oncoprotein dataset (detailed in the SOM file labeled, “SOM Table 4, source file”)

			Total beta *P*‐value	Oncoprotein beta *P*‐value
LUAD	Smoker	TCGA‐44‐2656	0.1202	0.0827
		TCGA‐44‐2659		
		TCGA‐44‐2668		
		TCGA‐44‐5644		
		TCGA‐44‐7670		
	Nonsmoker	TCGA‐44‐2665		
		TCGA‐50‐5066		
		TCGA‐86‐8279		
		TCGA‐86‐8585		
		TCGA‐86‐8672		
HNSC	Smoker	TCGA‐CN‐4723	0.5145	0.2851
		TCGA‐CR‐7402		
		TCGA‐CV‐6961		
		TCGA‐CV‐7245		
		TCGA‐D6‐6516		
	Nonsmoker	TCGA‐BA‐5152		
		TCGA‐BB‐4223		
		TCGA‐CR‐6472		
		TCGA‐CR‐6481		
		TCGA‐CV‐7252		
BLCA	Smoker	TCGA‐BT‐A2LB	0.0081	0.0100
		TCGA‐DKA1AC		
		TCGA‐DK‐A6AW		
		TCGA‐FD‐A6TC		
		TCGA‐ZF‐A9RC		
	Nonsmoker	TCGA‐DK‐A3WW		
		TCGA‐G2‐A2EO		
		TCGA‐K4‐A6FZ		
		TCGA‐MV‐A51V		
		TCGA‐SY‐A9G5		
PAAD	Smoker	TCGA‐3A‐A919	0.0476	0.0622
		TCGA‐H6‐8124		
		TCGA‐LB‐A7SX		
		TCGA‐OE‐A75W		
		TCGA‐PZ‐A5RE		
	Nonsmoker	TCGA‐2L‐AAQJ		
		TCGA‐3A‐A9J0		
		TCGA‐FB‐AAPU		
		TCGA‐HV‐A7OP		
		TCGA‐IB‐7652		

BLCA, bladder carcinoma; HNSC, head and neck squamous carcinoma; LUAD, lung adenocarcinoma; PAAD, pancreatic adenocarcinoma.

We found the BLCA cancer set had a significantly reduced level of methylation for smokers, in comparison with nonsmokers for both the average, overall genome methylation, and the average methylation of the genes for the oncoprotein set used above and defined in refs. (Fawcett et al. 2015; Parry et al. 2015) (*P* < 0.0085 and *P* < 0.011, respectively). The PAAD dataset also showed a significant difference in the average, overall genome methylation (*P* < 0.048), but did not indicate a difference for the oncoprotein set used in this study. And, no significant difference was found between nonsmokers and smokers in either the genome or oncoprotein gene methylation levels for the LUAD and HNSC datasets.

## Discussion

Specific cancer mutations have represented distinctions between nonsmoker and smoker cancers in previous studies (Albrecht and Theron 1988; Kondo et al. 1992), but this is the first direct, confirmation of an increased overall mutation rate in lung cancer that is attributed to smoking. Furthermore, the data above represent the first indication of a distinction in the number of CPCR mutations in lung and head and neck cancers, between nonsmoker and smoker cancers. Both of the preceding results are likely representative of a significant stochastic process of smoking‐dependent mutagenesis, keeping in mind that CPCR coding regions are relatively large and thus represent large mutagen targets.

The above work also indicates that the significant difference in CPCR mutations, between nonsmokers and smokers, leads to more deleterious amino acid substitutions among the smoker CPCR set. Because CPCR mutations are likely to have a dominant‐negative impact, consistent with their role in the formation of, and potential corruption of multimeric (polymer) cytoskeletal‐related structures, including the extracellular matrix, the increase in deleterious mutations may represent a proportional increase in the CPCR dysfunction in smoker cancers. The role of cytoskeleton and ECM dysfunction in cancer progression can be controversial, in that some reports indicate a requirement for cytoskeletal function for migration and other reports indicate a general association of dysfunction with metastasis and tumor aggressiveness (Pollack et al. 1968, 1980; Vogel et al. 1973; Kopelovich et al. 1977; Brinkley et al. 1980; Verderame et al. 1980; Chen et al. 1983; Zachary et al. 1986; Pokorna et al. 1994; Narumiya et al. 2009; Nurnberg et al. 2011; Carlier et al. 2013; Guo et al. 2013; Bear and Haugh 2014; Wang et al. 2014; Parry and Blanck 2016). This issue will not be settled here, but one possibility is that a certain level or type of cytoskeletal disruption remains consistent with, or even favors invadopodia formation and cell migration, while a distinct type of cytoskeletal disruption favors tissue detachment and circulation of tumor cells throughout the body.

While oncogene and tumor suppressor gene mutation rates distinguish nonsmokers and smokers in the LUAD dataset, no other TCGA dataset is represented by this distinction. Overall mutation rates and CPCR mutation rates distinguish nonsmoker and smoker HNSC groups, consistent with the large target afforded by the CPCR set that presumably registers the overall mutation rate. These data raise the question of whether nonsmoker and smoker cancers have phenotypic and clinical differences based on the greater level of CPCR mutations. These data also raise several related questions. Certain studies have indicated that higher cancer mutation rates indicate greater sensitivity to mutagenic drugs. This result could be due to reduced DNA repair functions or due to “weakened” cells unable to tolerate additional genotoxicity. Are there distinctions between nonsmoker and smoker responses to mutagenic chemotherapy? In addition, the above data would indicate that clinical differences dependent on oncogene or tumor suppressor gene mutations would not be apparent for nonsmoker and smoker cancers.

There are many possible explanations for cancer development besides mutations, including copy number variation, partial deletions, and epigenetic processes. In particular, certain studies have associated DNA methylation, or lack of methylation with cancers arising from tobacco use (Philibert et al. 2014; Bjaanaes et al. 2016). Data presented here do indicate an alternative explanation for a lack of mutation‐based distinction of nonsmoker and smoker cancers: demethylation of oncogenes in BLCA. These data are consistent with the possibility that nonsmoker and smoker pancreatic cancers are not distinguishable on the basis of mutation frequency.

Finally, the above data indicate that lung cancer arising after cessation of smoking represented reduced levels of overall mutations, the first such indication, although specific gene mutation profiles are known to be different in cancers arising years after smoking cessation (Bernardini et al. 2001; Ha and Califano 2002). The reduced number of mutations in smoking‐cessation cancers raises interesting questions about the change in the mutation profiles of the lung cells over the years since the use of tobacco. For example, are the heavily mutated cells indeed weakened and therefore lost to genotoxicity? Is there a natural rate of turnover in the cells that are sources of lung cancer, such that smoking‐cessation cancers are essentially the result of the same processes that lead to low mutation burdened, nonsmoker lung cancers?

## Conflict of Interest

None declared.
